# Tumor Microenvironment—A Short Review of Cellular and Interaction Diversity

**DOI:** 10.3390/biology11060929

**Published:** 2022-06-18

**Authors:** Aleksandra Bożyk, Kamila Wojas-Krawczyk, Paweł Krawczyk, Janusz Milanowski

**Affiliations:** Pneumonology, Oncology and Allergology Department, Medical University of Lublin, Jaczewskiego 8, 20-954 Lublin, Poland; kamilawojas@wp.pl (K.W.-K.); krapa@onet.pl (P.K.); janusz.milanowski@umlub.pl (J.M.)

**Keywords:** tumor microenvironment, immunophenotype, T lymphocytes

## Abstract

**Simple Summary:**

Scientists around the world have long been interested in understanding the biology of cancer. Numerous studies that have been carried out so far clearly show that the development of cancer stems not only from a genetic mutation, but also from a network of interconnections between chemical molecules and cells of various body systems. The complexity of the processes taking place in the tumor environment makes it extremely difficult to select a targeted treatment in patients with various types of cancer. Numerous scientific reports have revealed some of the interactions and processes responsible for tumor progression. In the era of molecularly targeted therapies and immunotherapy, this knowledge plays a key role in overcoming the mechanisms of cancer escape from immune system surveillance and in developing appropriate anti-cancer treatment.

**Abstract:**

The tumor microenvironment is a complex network of various interactions between immune cells and non-cellular components such as the extracellular matrix, exosomes and interleukins. Moreover, tumor heterogeneity and its constant modification may alter the immunophenotype and become responsible for its resistance regarding the therapies applied However, it should be remembered that in a strongly immunosuppressive neoplastic microenvironment, the immune system cells undergo reprogramming and most often cease to fulfill their original function. Therefore, understanding what happens within the tumor microenvironment, and which mechanisms are responsible for tumor development and progression should let us know how cancer could protect itself against the immune system. The presented review summarizes the latest information on the interactions between the tumor microenvironment and the cellular and non-cellular components, as well as their impact on cancer development, progression and immune system exhaustion.

## 1. Introduction

The development and formation of neoplastic cells is a phenomenon usually initiated by errors in the cell’s genome. Accumulation of genetic abnormalities ultimately results in the clinical manifestation of a tumor. However, it has been widely proven that there are several factors in the tumor’s microenvironment that promote and support its development. Moreover, tumor heterogeneity and its constant modifications, are also responsible for the resistance against the therapies applied. Within the tumor microenvironment, diversity of cells and intercellular interactions through different signals, such as: desmosomes, chemokines, cytokines and exosomes, are also all responsible for the host’s immune system’s activity suppression [1,2]. However, cancer has developed a number of immune surveillance escape mechanisms, making some tumors insensitive to targeted therapies. These include, inter alia, avoidance of recognition through the lack of expression of MHC class I (major histocompatibility complex) molecules, loss of antigenic epitopes, inactivation of immunological cells through the production of immunosuppressive factors, expression of anti-apoptotic proteins on cancer cells and a reduction in pro-apoptotic protein expression. A number of these mechanisms are conducive to progression, creation of their own environment for the development and closure of cells in their own favorable environment [3,4,5]. Therefore, understanding what happens within the tumor microenvironment and which mechanisms are responsible for tumor development and progression will let us know how cancer could protect itself against the immune system. Furthermore, identifying resistance mechanism to a given therapy is very important. The presented review summarizes the latest information regarding the interactions between tumor microenvironment components and their impact on cancer development, progression and immune system exhaustion.

## 2. What Happens When Neoplastic Cells Begin to Grow?

The tumor microenvironment consists of cellular, as well as non-cellular components including immune system cells. The essential cellular and non-cellular components are summarized in Table 1. The tumor microenvironment is a network of interactions between fibroblasts, vascular endothelial cells, pericytes, adipocytes and, most importantly, the cells of the immune response—T lymphocytes. Of note, tumor cells and stromal cells, during interaction, may change their immunophenotype with the progression of the tumor. Communication between the components of the microenvironment takes place through the interaction of signals produced by surrounding cells and the receptors on the surface of target cells. All the components of the tumor microenvironment are immersed in the extracellular matrix (ECM), which consists of cytokines, growth factors, enzymes, proteoglycans and glycoproteins. The presence of these components ensures the proper structure and homeostasis for communication and development of the neoplastic tissue. Therefore, it is important to understand the intercellular interactions, functions and mechanisms of their activation in order to inhibit the pathways leading to cancer progression [6,7,8].

One of the most important phenomena during the growth of a neoplastic cell is hypoxia, which seems to initiate functional changes in cells located in the tumor microenvironment [7,9]. First, a low oxygen level results in the strong proliferation of regulatory lymphocytes, thus inhibiting the differentiation of effector T lymphocytes [10]. Next, neoplastic cells begin to secrete the fibroblast growth factor (FGF), thus recruiting those cells into the tumor environment. Cancer-associated fibroblasts (CAF) contribute to production and creation of the neoplastic extracellular matrix, whose composition is poor in collagen [7]. Further, cancer-associated fibroblasts secrete growth factors and various immunosuppressive cytokines, such as CXCL1, CXCL2, CXCL3, CXCL12, CCL2, CCL5, CCL17, IL-8 and GM-CSF. All of those factors initiate angiogenesis and support tumor growth [11]. This results in creating unfavorable conditions and a specific inflammatory signal that stimulates the recruitment of immune system cells to the site of the neoplastic transformation. This influx applies to both specific (e.g., regulatory lymphocytes, T cells) and non-specific responsive cells (e.g., neutrophils, monocytes and myeloid cells). The chemokines and cytokines produced by cancer cells (mainly IL-6, IL-10 and TGF-β), have a huge systemic impact. The production of myeloid cells in the bone marrow is stimulated, which is intended to provide the cells necessary for the phagocytosis of foreign antigens. However, these cells cannot mature properly, they become immature myeloid-derived suppressor cells (MDSCs) that finally promote tumor growth and suppress immune system’s elements [7].

## 3. Diversity of Intercellular Interactions in the Tumor Microenvironment

**Figure 1:**
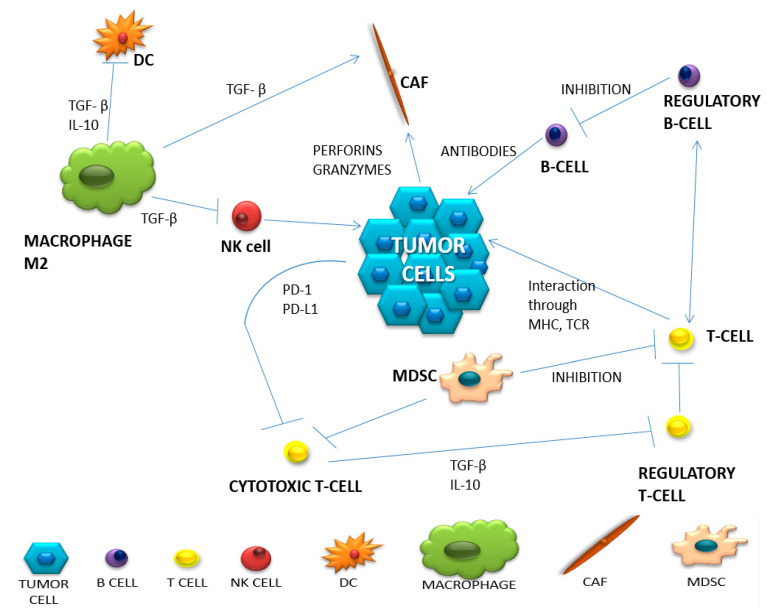
The network of intercellular interactions in the tumor microenvironment (based on Borros Arneth, 2019).

The appropriate action of the immune system is based on distinguishing the correct cells from the foreign cells and destroying the latter with the use of both mechanisms of its activity—specific and non-specific. However, it should be remembered that in a strongly immunosuppressive neoplastic microenvironment, the immune system cells undergo reprogramming and, most often, they no longer fulfill their original function [12]. A summary of the most important populations of immune system cells involved in the anti-cancer response and the change in their functioning in the neoplastic microenvironment is presented in Table 2. Complex intercellular interactions within the tumor microenvironment are presented in Figure 1.

The population of T lymphocytes is the main component of the specific immune response and within this group we can distinguish several subpopulations of T cells that are extremely important for the proper development of the immune response. The most important subpopulation of T lymphocytes during an anti-tumor response is one composed of cytotoxic T lymphocytes, which are designed to directly destroy the neoplastic cell after its specific recognition. Moreover, they produce a huge amount of proinflammatory cytokines (e.g., IL-2, INF-*γ*). Th1 lymphocytes arise when inactivated T lymphocytes are stimulated by IL-12 and express the transcription factor T-box, which induces the secretion of IFN- *γ* by Th1 lymphocytes. The main function of IFN-*γ* is an antitumor activity, stimulating cytotoxic T lymphocytes and NK cells. On the other hand, it has an inhibitory effect on Treg lymphocytes and recruits macrophages for their anti-tumor activity. In turn, by acting on dendritic cells, it stimulates MHC expression and antigen presentation [16]. Moreover, Th2 and Th17 helper lymphocytes secrete numerous cytokines (including IL-4, IL-5, IL-13, IL-17, IL-21 and IL-22) that contribute to increased tissue inflammation and thus stimulate tumor growth. Regulatory T cells, defined as cells positive for CD4 and CD25, and with the intracellular expression of FoxP3 protein, are one of the most important populations of lymphocytes responsible for extinguishing the activity of other immune system cells. An extremely important marker for this function is the CTLA-4 molecule that is induced on lymphocytes by TGF-beta released by tumor cells. Regulatory T lymphocytes inhibit the activity of other lymphocytes, mainly T cytotoxic ones, but also the ability of phagocytic cells. The mechanism of tryptophan consumption, which is another phenomenon of suppressing the immune response, should also be mentioned. Regulatory T lymphocytes use tryptophan, an amino acid essential for the functioning of cytotoxic T lymphocytes, to inhibit long-term inflammation. At the same time, they produce toxic products of metabolism such as hydrogen peroxide and nitric oxide (H_2_O_2_ and NO), disrupting the functioning of other immune cells. Another mechanism of inhibiting the function of immune cells is the production of adenosine by neoplastic cells during the process of ATP dephosphorylation due to prolonged inflammation. Outside the cell, in its presence, the expression of CD39 and CD73 increases and, having strong inhibitory properties, it leads to the disappearance of cytotoxic T lymphocytes, while stabilizing the activity of regulatory T lymphocytes [17]. In the context of different functions performed by selected populations of T lymphocytes, information, not only about the infiltration of the tumor site by the T cells, but also about the quality of this infiltration, seems to be extremely important. The cellular composition of the lymphocytic infiltration in the neoplastic tissue may be of good prognostic value [18]. The presence of CD8-positive cytotoxic T lymphocytes in the tumor environment is a positive prognostic factor in patients before starting treatment, because it plays one of the most important roles in the immune response [15,19].

Antigen recognition and antibody production are the main tasks of B lymphocytes. Even though B lymphocytes are known primarily for their stimulating role on the immune response, Ammirante et al. proved, in a mouse model of prostate cancer, that under the influence of the CXCL13 chemokine, they acquire a pro-tumor function [18,20,21]. In turn, under the influence of the produced cytokine IL-10 and the formation of complexes with IgG antibodies, they can promote the infiltration of immunosuppressive cells by destroying the ECM and stimulating angiogenesis. The presence of these cells in the tumor environment is associated with a good prognosis in patients with hepatocellular, biliary and breast cancer. In turn, in lung cancer, melanoma and pancreatic cancer, the presence of B lymphocytes worsens the prognosis of patients [7,22].

NK cells are attracted to the tumor by inflammation and chemokines in dendritic cells. Through perforins and granzymes, pro-inflammatory cytokines and chemokines (IFN-γ, IL-6, GM-CSF, CCL-5 and TNF), they are able to destroy foreign cells. However, their function in the tumor microenvironment is inhibited by impaired maturation, which means negligible expression of DX5, CD11b and CD27. For NK cells, the TIGIT receptor is an important checkpoint, which causes the blockade of the inhibitory pathway to restore their potent anti-tumor function [23].

Due to their cytotoxic and phagocytic nature, macrophages have long been considered as immunoactivating cells. However, the tumor microenvironment is such a complex structure that even macrophages undergo plastic modifications. Depending on the received signals, they modify phenotypes assuming pro-tumor functions (M2) or anti-tumor macrophages (M1). TAMs (tumor-associated macrophages) show defective activation of NF-κB in the presence of TNF-α and bacterial LPS (lipopolysaccharide) and maintain the inflammatory phenotype in the tumor. Some macrophages, under the influence of GM-CSF, IFN-γ and TNF-α, are transformed into the M1 macrophage population. Activated M1 macrophages stimulate cytotoxic T lymphocytes and NK cells, making them capable of killing cancer cells. However, the tumor microenvironment is usually infiltrated by M2 cells arising from the influence of IL-4, IL-10 and IL-13 [12,24].

Dendritic cells are known as the main antigen-presenting cells that stimulate T cell activity and their presence correlates with a good prognosis. There are several groups of a tumor infiltrating dendritic cells, which, depending on their function, may be either immunostimulatory or immunosuppressive. Among them, we can distinguish: plasmacytoid dendritic cells (pDCs), classical dendritic cells (cDCs 1 and 2) and monocyte-derived dendritic cells (moDCs). The main role of pDCs is to develop the body’s tolerance to cancer and its progression. They are divided into two types, depending on the presence of the CD2 antigen on the surface. They secrete IFN-α and –β; however, only cells with high CD2 expression, by secreting IL-12, promote proliferation of CD4-positive T cells. Another important group of dendritic cells are classical DCs, which can also be divided into two subgroups: cDC1 and cDC2. The first group is responsible for the secretion of inflammatory cytokines: IL-6, IL-8, IL-12 and TNF-α. In turn, cDC2 constitutes a large percentage of the entire population of dendritic cells, demonstrating high efficiency in antigen presentation and increasing the CD4-positive T cell population. Therefore, their presence in the tumor microenvironment suggests the generation of an anti-tumor response. The last subpopulation of dendritic cells are monocytes transformed into inflammatory dendritic cells. They show similar expression of surface antigens, as in the case of cDC2s. The presence of this population in lung cancer has been proven [25]. In inflammation, dendritic cells can start producing TNF and nitric oxide (NO), which is essential for CD8-positive T cells to perform an anti-cancer function. However, neoplastic cells secreting CXCL1, CXCL5, CCL2 and VEGF inhibit dendritic cell maturation, with the side effect of changing the function of a dendritic cell into a pro-neoplastic cell [17,26].

Neutrophils, in a properly functioning organism, perform a defensive function, mainly through the phagocytosis of dead cells and as antigen-presenting cells. In the cancer microenvironment, tumor-associated neutrophils (TANs) secret various cytokines and metabolic products (myeloperoxidase—MPO), therefore they influence the recruitment of monocytes and macrophages, providing them with pro- or anti-tumor functions. Under the influence of TGF-β, neutrophils can transform into having a pro-neoplastic function (N2 subtype), and they can stimulate angiogenesis, while IFN-γ promotes the anti-tumor N1 subtype. This subtype has an antitumor character which is manifested by the possibility of phagocytosis and by bringing foreign cells into apoptosis. The presence of neutrophils in the neoplastic microenvironment lowers the prognostic value of the disease [7,24,27].

Adipocytes are the main component of adipose tissue, where they act as an energy reservoir. In the tumor microenvironment, they secrete a multitude of growth factors, hormones and cytokines. They fulfill their mainly pro-neoplastic function by secreting adipokines, which include leptin and the hepatocyte growth factor, causing inflammation and increasing the likelihood of metastasis. On the other hand, excessive stimulation of adipocytes contributes to the growth of collagen and stiffening of the structure of the microenvironment [14].

Cancer-associated fibroblasts (CAFs) are the main source of collagen for cells, they affect the surrounding tumor, immune and endothelial cells. Their presence contributes to resistance to therapies and more frequent relapses of many neoplastic diseases, and is associated with a poor prognosis. They secrete many cytokines and growth factors such as: TGF-*β*, IL-6, exosomes, CXCL2, CCL7, HGF, IGF and CTGF. It has been proven that CAFs promote tumor development by causing inflammation, stimulation of the angiogenesis process, secretion of growth factors and modification of ECM [28,29,30].

In breast cancer, adipocytes are the main ingredients that build the tumor microenvironment. Cancer-associated adipocytes (CAA) are characterized by a smaller size, they secrete more chemokine ligand 2 and 5 (CCL2, CCL5), IL-1β, IL-6, leptin, VEGF and TNF-α, but lower the expression of adiponectin. These are largely responsible for storing energy in the form of triacylglycerols to be released as free fatty acids when needed. CAAs are also able to influence the functions of immune cells through the release of pro-inflammatory cytokines IL-6, IL-8 and TNF-α. Thus, they attract monocytes and macrophages, creating chronic inflammation. As a result of lipolysis, free fatty acids are released, which disturbs lipid homeostasis and also influences the maturation of cells of the immune system [31,32,33].

The cells strongly influencing the suppression of immune cells in the tumor microenvironment are myeloid-derived suppressor cells (MDSC). They come from the bone marrow, inhabiting the peripheral lymph nodes or the tumor microenvironment. Depending on the target location, they could perform different functions. In TME, they support neoplastic growth, metastases and participate in angiogenesis. There are two types of MDSC: M-MDSC (monocytic-MDSC) and PMN-MDSC (polymorphonuclear-MDSC). In TME, M-MDSC transforms into TAM (tumor-associated macrophages). This shows the complexity in the formation and functioning of the neoplastic microenvironment. Additionally, the complex network of cellular interactions may make it difficult to respond to treatment [34]. An example of this is a recent study in mice that were administered a STAT3 inhibitor. The observations made showed a reduction in the amount of MDSC in the spleen, while it remained unchanged in the tumor [35].

## 4. Three Categories of Tumor Microenvironments Based on Their Immunophenotype

Based on the presence and the strength of the immune system invasion into the tumor site, neoplastic tumors may have different immunophenotypes. The following immunoprofiles, based on the activation and infiltration of the immune system, could distinguish: (1) “hot” tumors, which are strongly infiltrated by T lymphocytes and with many inflammatory signals; (2) “cold” tumors, which are scant of any immune cell infiltration nor inflammatory signs; (3) tumors with immune exclusion, where immune cells are at the periphery or within the stromal tissue.

The infiltrated tumors, so called “hot”, inflammatory tumors, are defined by strong leukocyte infiltration, including a huge diversity of cells: B lymphocytes, CD4-positive and CD8-positive T cells, Treg lymphocytes, macrophages, fibroblasts and MDSC. Additionally, these tumors are characterized by the presence of intra-tumor chemokines (e.g., CXCL9, CXCL10, CCL5) [10]. IFN-γ is also released, which, although it has an anti-tumor effect, stimulates the immunosuppressive response by induction of IDO (indole 2,3-dioxygenase) and PD-L1 expression. It has also been proven that the presence of these molecules indicates the presence of cytotoxic T lymphocytes [10,36,37]. In this type of tumor, there are many interactions between negative immune checkpoints on effector T cells and their ligands on APC and tumor cells. The main signal stimulating T cells to activate is the association of CD28 on their surface with CD80 and CD86 on tumor cells or APC. However, when CD28 binds to CTLA-4 on a cancer cell (which has a greater affinity for CD28 than CD80 or CD86), T cells become depleted, anergic and killed. The “hot” tumors are associated with denser PD-1-positive T lymphocyte infiltration, with a pre-existing primed immune response and are more likely to respond to the anti-PD-1 or anti-PD-L1 blockade used as monotherapy.

**Figure 2:**
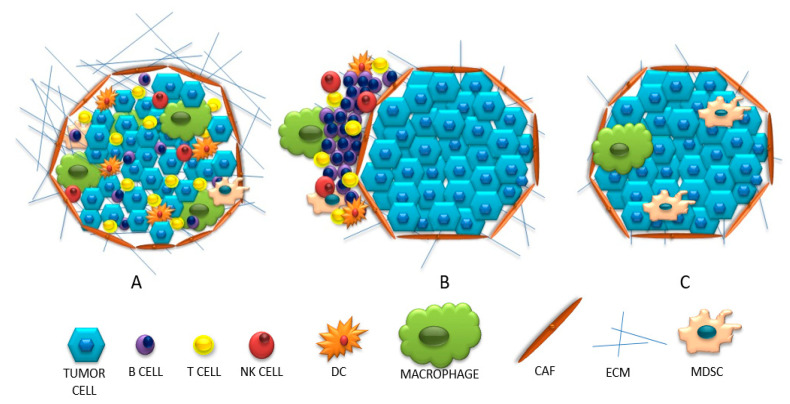
(**A**) “Hot” (inflamed), (**B**)”cold”(excluded), (**C**) “cold “(ignored) tumors.

“Cold” (non-inflammatory) tumors can be divided into two types: the ones with invasion of immune cells and the so-called “immune desert”. The first group is characterized by an influx of cells in the vicinity of the tumor without penetrating its center. It is suspected that APCs may play a role here; in the absence of stimulation, they inhibit the influx of T lymphocytes into the center of the tumor or release an insufficient amount of chemokines recruiting T lymphocytes [18,37]. The second concept for this behavior of lymphocytes is the inability to pass the barrier surrounding the tumor parenchyma (caused, for example, by a compact network of collagen fibers) [18]. Therefore, in this type of tumor, little or no intercellular interactions are observed due to the ‘barrier character ‘of fibroblast cells. Whereas the second group describes a condition in which there is no infiltration or activation of T lymphocytes, with the presence of Treg lymphocytes, macrophages and MDSC. These cells inhibit the maturation of dendritic cells and make it difficult for T cells to be infiltrated. When the amount of adhesion molecules (CD34, E-selectin, vascular cell adhesion molecule—VCAM and intercellular adhesion molecule—ICAM) is reduced under the influence of VEGF and FGF, VEGF stimulates blood vessel formation and reduces cell adhesion. In turn, the stimulated ligand for the Fas receptor (FasL) inhibits the influx of T lymphocytes [37]. The presence of IDO, TGF-β and PD-L1 molecules in the microenvironment is not conducive to the survival and activation of T lymphocytes, but may additionally stimulate MDSC cells. These, using arginine, reduce the survival of T lymphocytes. This condition is called endothelial cell anergy, which results in the inability to adhere T lymphocytes in the tumor blood vessel and its infiltration [18,36,37,38]. A comparison of the discussed immunophenotypes is presented in Figure 2.

## 5. The Tumor Microenvironment in Melanoma, Breast and Kidney Cancer

Each neoplastic tumor presents certain differences distinguishing it from other neoplasms, which are characteristic only of them. One such feature is the presence of various cells infiltrating the tumor microenvironment. In breast cancer, macrophages are the most abundant population of immune cells. The presence of mature dendritic cells is associated with a lower number of metastases and a better prognosis. The opposite is true in the melanoma microenvironment as here, dendritic cells indicate a poor prognosis of the disease. In turn, the predominance of cytotoxic T lymphocytes and neutrophils predisposes to a better course of the disease. In addition, renal cell carcinoma is the most infiltrated cancer among kidney cancers. It exhibits the highest percentage of cytotoxic and Th1 helper T lymphocytes, neutrophils and DC, while a small percentage of regulatory T lymphocytes and Th2 lymphocytes are observed, which is a pro-inflammatory nature of the tumor microenvironment. A high level of cytotoxic T lymphocytes is associated with a worse prognosis for the patient, which indicates that these cells are suppressed and dysfunctional. Additionally, the presence of macrophages and stromal cells may indicate a worse prognosis for the patient [39,40,41].

## 6. The Use of Cellular Interactions in the Treatment of Cancer

Any activity of the immune system must be balanced and regulated. Too long a duration of excitation, as a result of, for example, chronic inflammation, may result in the appearance of autoantibodies. The discovery of the mechanisms of regulating the body’s immune response in the 1990s, contributed to the spectacular development of a new method of treating cancer patients with immunotherapy. On the surface of T lymphocytes, which are mainly responsible for the development of the immune response, there are immune checkpoint receptors that stabilize the body’s response to foreign antigens. Depending on their function, these receptors can be divided into two types: costimulatory receptors (CD27, CD28, CD137, OX40 (CD134), GITR (glucocorticoid-induced TNF receptor) and inhibitory ones (CTLA-4, PD-1, TIM- 3, LAG3, BTLA). In this case, stimulation of these receptors leads to lymphocyte anergy, suppression of the immune response and development of immunosuppression [9,33,34]. The most popular molecule used in cancer therapy is PD-1 and its ligand PD-L1. The programmed cell death receptor PD-1 is located on T lymphocytes, NK cells and unstimulated B lymphocytes. Its task is to send a signal that inhibits the activity of T lymphocytes, thereby impairing the normal immune response.

A suitable ligand for this role, PD-L1, is a transmembrane glycoprotein found on the islets of the pancreas, retina and placenta. As a protein, PD-L1 is localized on the surface of B and T lymphocytes, APC (antigen-presenting cell) cells, dendritic cells and monocytes.

However, the continuous exposure of neoplastic cells to IFN ɣ may stimulate them to change their immune profile and develop a mechanism of escape from immune control. It is also not uncommon to see specific surface molecules appear on the surface of neoplastic cells, blocking the functions of the immune system cells. An example of such a defense mechanism is the PD-L1 molecule on a cancer cell. When it binds to the PD-1 molecule on the T lymphocyte, PD-L1 sends an inhibitory signal, leading to T-lymphocyte anergy. The purpose of immunotherapy is, among other things, to block the conduction of the inhibitory signal and thus stimulate T lymphocytes to destroy cancer cells using the anti-PD-1 monoclonal antibodies (nivolumab, pembrolizumab) or anti-PD-L1 (atezolizumab, durvalumab, avelumab) [42,43,44,45,46].

There are also numerous clinical trials testing monoclonal antibodies that block targets other than PD-1 and PD-L1 negative immune checkpoints. LAG3 and TIGIT are the most widely tested molecules, and anti-TIGIT monoclonal antibodies are used in the clinic in combination therapy with anti-PD-L1 immunotherapy. In addition, not only negative checkpoint blocking antibodies, but also positive checkpoint agonist antibodies are being tested in numerous clinical studies. An example is testing using anti-CD27 or anti-CD137 [47,48]. Currently, research is also underway on the use of anti-IDO antibodies and antibodies blocking the adenosine a2a receptors, which can potentially increase the effectiveness of the currently used therapies [49,50].

## 7. Conclusions

Understanding the components and intercellular interactions in the tumor microenvironment is the first step to recognizing the mechanisms of progression and the resistance to treatment. The tumor microenvironment is a complex network of connections between cellular and non-cellular components. Regulation of intercellular interactions in the tumor microenvironment concerns not only the participation of cytokines, chemokines and metabolic products, but also the participation of epigenetic factors, including microRNA activity (e.g., miR-31, miR-125, miR-520); in addition, the phenomenon of methylation DNA and histone modification are of great importance. The search for microsatellite instability (MSI) is also important in predicting the response to therapy in patients with gastric and colorectal cancers. It has been proven that, in every population (White, Asian or Black), high microsatellite instability is significantly associated with a better disease prognosis [51]. In the population of CRC in India, a lower percentage of mutations in the *p-53* gene was demonstrated, while *K-ras* and *APC* alterations occurred with a similar frequency as in the Western population [52]. However, in order to propose a correct and professional analysis of the available knowledge in the above-mentioned topics, another extensive study should be undertaken. The constantly expanding knowledge in the field of cancer biology makes us realize how complicated the process of cancer formation is. In order to inhibit its development, a multidisciplinary and comprehensive approach is needed to understand the pathways of intercellular interaction, which is the secret of successful cancer immunotherapy.

## Figures and Tables

**Table 1:** The essential cellular and non-cellular components of the tumor microenvironment [6,7,8].

Cellular Components	Non-Cellular Components
T lymphocytes	Extra-cellular matrix (ECM)
B lymphocytes	IFN *γ*
Macrophages	Tumor necrosis factor (TNF)
Adipocytes	Growth factors (PDGF, EGF, NGF, TGF*α, β*)
Neutrophils	IL-1, IL-2, IL-3, IL-4, IL-5, IL-6, IL-12, IL-15, IL-18, IL-37, IL-23, IL-27, IL-7, IL-37, IL-31, IL-10
Cancer associated fibroblasts (CAF)	Chemokines (MIP, CCL11, CCL5, MCP, IL-8, IL-16, CCL9)
Endothelial cells	Exosomes
Cancer cells	Apoptotic bodies
NK cells	
Dendritic cells (DC)	

**Table 2:** Different cell populations in the tumor microenvironment [7,13,14,15].

Cell Type	Natural Function	Function in Tumor Microenvironment	Produced Substances	
			of Anti-Tumor Activity	of Pro-Tumor Activity
HELPER T CELLS	Th1-stimulates dendritic and NK cells. Attracts T lymphocytes.	Th1- blocked by IL-4, IL-10, TGF-*β*, Treg, Th2, M2 Th2-. Inhibition of Th1. Stimulation of M2 population of macrophages.	TNF-α, IL-12, IL-17,IL-18, IL-21, IL-27.	Th17 lymphocytes can transform into Treg lymphocytes.
REGULATORY T CELLS	Protection against autoimmunity from autoreactive T lymphocytes.	Stimulating immune tolerance. Immunosuppression. Inhibition of the immune response.	IL-10, TGF-*β,* adenosine, PGE2, IL-35.	TGF-*β*, IL-2, IL-10, IL-35.
CYTOTOXIC T CELLS	Cytotoxic. Stimulation of other immune cells to infiltrate the tumor.	Inhibition of the cytotoxic function by binding to PD-L1.	Perforins. Granzymes IL-2, TNF-α, IFN-*γ*.	------
MACROPHAGES	Destruction, phagocytosis of abnormal cells. Inducing inflammation.	TAM2: Protumor. Inhibition of the inflammatory process.	IFN-*γ*, IL-12, GM-CSF.	IL-10, TGF-β, EGF, FGF, VEGF, MMP CCL2, CCL5, CCL3, CCL8, CCL22.
NEUTROPHILS	Phagocytosis. ADCC. Stimulation of CD8 + lymphocytes, NK cells.	N1: Phagocytosis. Stimulation of apoptosis N2: Angiogenesis. Stimulation of the inflammatory process in the tumor.	TNF-*α*, IFN- *γ*.	TGF-*β*, MPO, MMP9, HGF, VEGF
B CELLS	Presenting antigens. Complement activation. Antibody production.	Supporting angiogenesis. -inhibition of anti-cancer activities. Stimulation of Treg.	IL-2.	IL-10, TGF-*β*.
CANCER ASSOCIATED FIBROBLASTS (CAF)	--------	Secreting growth factors. Causing inflammation.	--------	CXCL1, CXCL2, CXCL3, CXCL12, CCL2, CCL5, CCL17, IL-8, GM-CSF TGF-*β*, IL-6, exosomes, HGF, IGF, CTGF.
NATURAL KILLER CELLS (NK)	Cytotoxic. Immune supervision. Stimulation of T and DC lymphocytes.	--------	IL-2, IL-6, 12, 15, IFN-*γ*, TNF-*α*, GM-CSF, CCL-5.	--------
DENDRITIC CELLS (DC)	Presentation of antigens. Influences the differentiation of helper and regulatory lymphocytes. Cytotoxic function.	Presence on the surface of PD-L1, Impaired antigen presentation, maturation and tumor infiltration.	IL-6, IL-8, IL-12, IL-15.	--------
CANCER ASSOCIATED ADIPOCYTES (CAA)	Production and storage of simple fats (triglycerides).	Secretion of adipokines. They cause changes in the metabolism of cancer cells and remodel the ECM.	--------	Adipokines: leptin, hepatocyte growth factors IL-1*β*.
MYELOID-DERIVED SUPPRESSOR CELLS (MDSC)	--------	Suppression of the immune response.	--------	IL-4, CCL3, CCL4, CCL5, PGE2, NO.

IL, interleukin; Th1, helper T lymphocyte 1; Th2, helper T lymphocyte 2; TGF-β, transforming growth factor beta; TNF-α, tumor necrosis factor alfa; PGE2, prostaglandin E2; PD-L, programmed death cell ligand 1; TAM, tumor-associated macrophages; IFN-γ, interferon gamma; GM-CSF, granulocyte-macrophage colony-stimulating factor; EGF, epidermal growth factor; FGF, fibroblast growth factor; VEGF, vascular endothelial growth factor; MMP, matrix metalloproteinases; CCL, chemokines; ADCC, antibody-dependent cell cytotoxicity; MPO, myeloperoxidase; HGF, hepatocyte growth factor; IGF, insulin-like growth factor; CTGF, connective tissue growth factor; ECM, extracellular matrix; NO, nitric oxide.

## Data Availability

No new data were created or analyzed in this study. Data sharing is not applicable to this article.

## References

[1] Li I., Nabet B.Y. (2019). Exosomes in the tumor microenvironment as mediators of cancer therapy resistance. Mol. Cancer.

[2] Hinshaw D.C., Shevde L.A. (2019). The tumor microenvironment innately modulates cancer progression. Cancer Res..

[3] Zhou J., Rao L., Yu G., Cook T.R., Chen X., Huang F. (2021). Supramolecular cancer nanotheranostics. Chem. Soc. Rev..

[4] Zhou J., Yu G., Huang F. (2017). Supramolecular chemotherapy based on host-guest molecular recognition: A novel strategy in the battle against cancer with a bright future. Chem. Soc. Rev..

[5] Ding Y., Tong Z., Jin L., Ye B., Zhou J., Sun Z., Mao Z. (2022). An NIR Discrete Metallacycle Constructed from Perylene Bisimide and Tetraphenylethylene Fluorophores for Imaging-Guided Cancer Radio-Chemotherapy. Adv. Mater..

[6] Baghban R., Roshangar L., Jahanban-Esfahlan R., Seidi K., Ebrahimi-Kalan A., Jaymand M., Zare P. (2020). Tumor microenvironment complexity and therapeutic implications at a glance. Cell Commun. Signal.

[7] Farc O., Cristea V. (2020). An overview of the tumor microenvironment, from cells to complex networks (Review). Exp. Ther. Med..

[8] Tamminga M., Hiltermann T.J.N., Schuuring E., Timens W., Fehrmann R.S.N., Groen H.J.M. (2020). Immune microenvironment composition in non-small cell lung cancer and its association with survival. Clin. Transl. Immunol..

[9] Petrova V., Annicchiarico-Petruzzelli M., Melino G., Amelio I. (2018). The hypoxic tumour microenvironment. Oncogenesis.

[10] De Guillebon E., Dardenne A., Saldmann A., Séguier S., Tran T., Paolini L., Tartour E. (2020). Beyond the concept of cold and hot tumors for the development of novel predictive biomarkers and the rational design of immunotherapy combination. Int. J. Cancer.

[11] Wang M., Zhao J., Zhang L., Wei F., Lian Y., Wu Y., Guo C. (2017). Role of tumor microenvironment in tumorigenesis. J. Cancer.

[12] Gołąb J., Jakóbisiak M., Lasek W., Stokłosa T. (2017). Immunologia.

[13] Deng S., Clowers M.J., Velasco W.V., Ramos-Castaneda M., Moghaddam S.J. (2020). Understanding the Complexity of the Tumor Microenvironment in K-ras Mutant Lung Cancer: Finding an Alternative Path to Prevention and Treatment. Front. Oncol..

[14] Pallegar Nikitha K. (2020). CSL. Adipocytes in the Tumour Microenvironment. Adv. Exp. Med. Biol..

[15] Iwahori K. (2018). Cytotoxic CD8+ Lymphocytes in the Tumor Microenvironment. Adv. Exp. Med. Biol..

[16] Lee J., Lozano-Ruiz B., Yang F.M., Fan D.D., Shen L., González-Navajas J.M. (2021). The Multifaceted Role of Th1, Th9, and Th17 Cells in Immune Checkpoint Inhibition Therapy. Front. Immunol..

[17] Lei X., Lei Y., Li J.K., Du W.X., Li R.G., Yang J., Tan H.B. (2020). Immune cells within the tumor microenvironment: Biological functions and roles in cancer immunotherapy. Cancer Lett..

[18] Galli F., Aguilera J.V., Palermo B., Markovic S.N., Nisticò P., Signore A. (2020). Relevance of immune cell and tumor microenvironment imaging in the new era of immunotherapy. J. Exp. Clin. Cancer Res..

[19] Jeske S.S., Weissinger S.E., Veit J.A., Brunner C., Huber U., Theodoraki M.N., Doescher J. (2019). Treatment-induced changes of lymphocyte subsets in patients with adenoid cystic carcinoma of the head and neck. Eur. Arch. Oto-Rhino-Laryngol..

[20] Massimo A., Jun-Li L., Sergei G., Sergei N., Michael K. (2017). Prostate Cancer—NCCN Evidence Blocks. Version 2.2017. Am. Cancer Soc..

[21] Borros A. (2020). Tumor microenvironment. Medicina.

[22] Tsou P., Katayama H., Ostrin E.J., Hanash S.M. (2016). The emerging role of b cells in tumor immunity. Cancer Res..

[23] Qing Z., Jiacheng B., Xiaodong Z., Yongyan C., Hua W., Wenyong W., Zhengguang W., Wu Q., Peng H., Wei H. (2018). Blockade of the checkpoint receptor TIGIT prevents NK cell exhaustion and elicits potent anti-tumor immunity. Nat. Immunol..

[24] Kim J., Bae J.S. (2016). Tumor-associated macrophages and neutrophils in tumor microenvironment. Mediators Inflamm..

[25] Zilionis R., Engblom C., Pfirschke C., Savova V., Zemmour D., Saatcioglu H.D., Klein A.M. (2019). Single cell transcriptomics of human and mouse lung cancers reveals conserved myeloid populations across individuals and species. Immunity.

[26] Verneau J., Sautés-Fridman C., Sun C.M. (2020). Dendritic cells in the tumor microenvironment: Prognostic and theranostic impact. Semin. Immunol..

[27] Wu Lingyun S.S., Singh Rakesh K. (2020). Neutrophils in the tumor microenvironment. Adv. Exp. Med. Biol..

[28] Ishii G., Ochiai A., Neri S. (2016). Phenotypic and functional heterogeneity of cancer-associated fibroblast within the tumor microenvironment. Adv. Drug Deliv. Rev..

[29] Monteran L., Erez N. (2019). The dark side of fibroblasts: Cancer-associated fibroblasts as mediators of immunosuppression in the tumor microenvironment. Front Immunol..

[30] LeBleu V.S., Kalluri R. (2018). A peek into cancer-associated fibroblasts: Origins, functions and translational impact. DMM Dis. Model Mech..

[31] Wu Q., Li B., Sun S., Sun S. (2020). Unraveling Adipocytes and Cancer Links: Is There a Role for Senescence?. Front. Cell Dev. Biol..

[32] Wu Q., Li B., Li Z., Li J., Sun S., Sun S. (2019). Cancer-associated adipocytes: Key players in breast cancer progression. J. Hematol. Oncol..

[33] Wu Q., Li B., Li J., Sun S., Yuan J., Sun S. (2021). Cancer-associated adipocytes as immunomodulators in cancer. Biomark. Res..

[34] Kumar V., Patel S., Tcyganov E., Gabrilovich D.I. (2016). The Nature of Myeloid-Derived Suppressor Cells in the Tumor Microenvironment. Trends Immunol..

[35] Kumar V., Cheng P., Condamine T., Mony S., Languino L.R., McCaffrey J.C., Gabrilovich D.I. (2017). CD45 Phosphatase Inhibits STAT3 Transcription Factor Activity in Myeloid Cells and Promotes Tumor-Associated Macrophage Differentiation. Physiol. Behav..

[36] Duan Q., Zhang H., Zheng J., Zhang L. (2020). Turning Cold into Hot: Firing up the Tumor Microenvironment. Trends Cancer.

[37] van der Woude L.L., Gorris M.A.J., Halilovic A., Figdor C.G., de Vries I.J.M. (2017). Migrating into the Tumor: A Roadmap for T Cells. Trends Cancer.

[38] Ros X.R., Vermeulen L. (2018). Turning Cold Tumors Hot by Blocking TGF-β. Trends Cancer.

[39] Soysal S.D., Tzankov A., Muenst S.E. (2015). Role of the Tumor Microenvironment in Breast Cancer. Pathobiology.

[40] Yang S., Liu T., Nan H., Wang Y., Chen H., Zhang X., Liang G. (2020). Comprehensive analysis of prognostic immune-related genes in the tumor microenvironment of cutaneous melanoma. J. Cell Physiol..

[41] Vuong L., Kotecha R.R., Voss M.H., Hakimi A.A. (2019). Tumor microenvironment dynamics in clear-cell renal cell carcinoma. Cancer Discov..

[42] Wojas-Krawczyk K., Krawczyk P. (2015). Rozwój koncepcji przeciwnowotworowej immunoterapii. Onkol. W Prakt. Klin..

[43] Wojas-Krawczyk K., Kubiatowski T. (2020). Imperfect predictors for lung cancer immunotherapy—A field for further research. Front Oncol..

[44] Topalian S.L., Taube J.M., Anders R.A., Pardoll D.M. (2016). Mechanism-driven biomarkers to guide immune checkpoint blockade in cancer therapy. Nat. Rev. Cancer.

[45] Herbst R.S., Baas P., Kim D.-W., Felip E., Pérez-Gracia J.L., Han J.-Y., Garon E.B. (2016). Pembrolizumab versus docetaxel for previously treated, PD-L1-positive, advanced non-small-cell lung cancer (KEYNOTE-010): A randomised controlled trial. Lancet.

[46] Grzywnowicz M., Giannopoulos K. (2012). W Układzie Immunologicznym Oraz Nowotworach. Acta Haematol. Pol..

[47] Buchan S.L., Fallatah M., Thirdborough S.M., Taraban V.Y., Rogel A., Thomas L.J., Al-Shamkhani A. (2018). Pd-1 blockade and cd27 stimulation activate distinct transcriptional programs that synergize for CD8þ T-cell–driven antitumor immunity. Clin. Cancer Res..

[48] Chu D.T., Bac N.D., Nguyen K.H., Tien N.L.B., Van T.V., Nga V.T., Thimiri Govinda Raj D.B. (2019). An update on anti-CD137 antibodies in immunotherapies for cancer. Int. J. Mol. Sci..

[49] Tang K., Wu Y.H., Song Y., Yu B. (2021). Indoleamine 2, 3-dioxygenase 1 (IDO1) inhibitors in clinical trials for cancer immunotherapy. J. Hematol. Oncol..

[50] Carpenter B., Lebon G. (2017). Human adenosine A2A receptor: Molecular mechanism of ligand binding and activation. Front Pharmacol..

[51] Cai L., Sun Y., Wang K., Guan W., Yue J., Li J., Wang L. (2020). The Better Survival of MSI Subtype Is Associated With the Oxidative Stress Related Pathways in Gastric Cancer. Front Oncol..

[52] Malhotra P., Anwar M., Nanda N., Kochhar R., Wig J.D., Vaiphei K., Mahmood S. (2013). Alterations in K-ras, APC and p53-multiple genetic pathway in colorectal cancer among Indians. Tumor Biol..

